# The association between telemedicine and patient outcomes in osteoarthritis and inflammatory arthritis: a systematic review and meta-analysis of randomized controlled trials

**DOI:** 10.3389/fmed.2026.1897145

**Published:** 2026-10-14

**Authors:** Yize Zhang, Meijie Zhou, Fangzhen Wu, Yanfen Guo, Xiaorong Lin, Zhongwen Zhao

**Affiliations:** 1 Second Clinical Medical College, Fujian University of Traditional Chinese Medicine, Fuzhou, Fujian, China; 2 School of Public Health, Hainan Medical University, Haikou, Hainan, China; 3 Department of Rheumatology and Endocrinology, The Second Affiliated Hospital of Fujian University of Traditional Chinese Medicine, Fuzhou, Fujian, China

**Keywords:** inflammatory arthritis, meta-analysis, osteoarthritis, systematic review, telemedicine

## Abstract

**Background:**

Osteoarthritis (OA) and inflammatory arthritis (IA) are prevalent chronic musculoskeletal disorders that cause persistent pain, functional impairment, and reduced quality of life. Telemedicine offers a potential strategy for long-term arthritis management, but the strength of the supporting evidence remains uncertain. This systematic review and meta-analysis evaluated the efficacy of telemedicine interventions in patients with OA and IA.

**Methods:**

We searched PubMed, Embase, Cochrane Library, Web of Science, and WHO ICTRP up to 10 July 2026 for randomized controlled trials (RCTs). Two reviewers independently conducted study selection, data extraction, and risk-of-bias assessment using the RoB 2.0 tool. Random-effects meta-analysis was performed to calculate standardized mean differences (SMDs) with 95% confidence intervals (CIs) and 95% prediction intervals (PIs). The certainty of evidence was rated using the GRADE framework.

**Results:**

Twenty-four RCTs enrolling 2,262 patients (1,568 with OA and 694 with IA) were included. Telemedicine was associated with improvements in pain (SMD = −0.66, 95% CI −1.00 to −0.32; 95% PI −1.90 to 0.58), quality of life (SMD = 0.42, 95% CI 0.12 to 0.71; 95% PI −0.60 to 1.44), WOMAC scores (SMD = −0.78, 95% CI −1.19 to −0.37; 95% PI −2.25 to 0.69), and self-efficacy (SMD = 0.47, 95% CI 0.06 to 0.89; 95% PI −1.04 to 1.98). All primary outcomes showed substantial heterogeneity (*I*^2^ > 75%), with 95% PIs that spanned the null value, indicating that the true effect in future studies could be zero or even negative. The self-efficacy finding lacked robustness in leave-one-out sensitivity analyses and was susceptible to the influence of individual studies. Evidence for IA was restricted to a small number of trials on rheumatoid arthritis and ankylosing spondylitis, and the overall certainty of evidence was rated very low to low.

**Conclusions:**

Current evidence suggests an association between telemedicine and improved outcomes in arthritis, but these findings are exploratory and limited by low evidence certainty, marked heterogeneity, and wide prediction intervals. Evidence for IA is insufficient to draw definitive conclusions.

**Systematic review registration:**

https://www.crd.york.ac.uk/PROSPERO/view/CRD420261401824, PROSPERO CRD420261401824.

## 1 Introduction

Osteoarthritis (OA) and inflammatory arthritis (IA) are common chronic musculoskeletal conditions associated with substantial morbidity and impaired quality of life. OA, the most prevalent form of arthritis, commonly affects the knee, hip, and hand joints (1), whereas IA encompasses inflammatory rheumatic diseases such as rheumatoid arthritis (RA) and ankylosing spondylitis (AS). Patients with OA or IA commonly experience pain, stiffness, swelling, impaired mobility, and, in some forms of IA, extra-articular manifestations, which may contribute to long-term disability and reduced quality of life (1, 2). According to the Global Burden of Disease (GBD) study, about 595 million people worldwide had OA in 2020, and about 17.6 million people had RA. The prevalence and absolute burden of both conditions are projected to increase further by 2050 (3, 4). Accordingly, OA and IA represent substantial and growing challenges for both clinical care and healthcare systems.

Current OA management guidelines emphasize patient education, exercise, weight management, pharmacological treatment, and self-management as key components of long-term care (5). Management of IA additionally requires early diagnosis, timely initiation and adjustment of pharmacological therapy, regular monitoring of disease activity, and sustained patient involvement in self-management (6, 7). However, conventional follow-up remains largely dependent on in-person healthcare visits, which may impose substantial time and financial burdens and limit access to continuous care, particularly for patients requiring repeated assessment and long-term disease management.

Telemedicine enables the remote delivery of healthcare services through digital communication technologies when patients and healthcare professionals are geographically separated (8). It can support clinical consultation, treatment delivery, monitoring, rehabilitation, and long-term disease management (9). With the rapid expansion of digital health technologies, telemedicine has become an increasingly important approach to improving access to care and supporting the long-term management of chronic diseases (10). Previous systematic reviews have suggested that telemedicine interventions may improve pain, physical function, and self-efficacy in patients with OA (11, 12). Similar approaches have also been associated with improvements in disease activity, physical function, and self-management in patients with IA (13, 14). However, the available evidence remains fragmented across disease populations and telemedicine modalities. Studies also vary substantially in intervention type, duration, comparator conditions, and outcome assessment, limiting the consistency and interpretability of the available evidence.

Recently, Zhu et al. conducted a systematic review and meta-analysis of telemedicine-based exercise interventions in older adults with OA. Their study found that these interventions improved pain, physical function, and quality of life (15). However, that review focused only on older adults with OA and primarily assessed exercise-based telerehabilitation interventions. Therefore, its findings may not be directly applicable to patients with IA and may not fully reflect the broader range of telemedicine-based self-management interventions. Accordingly, the present systematic review and meta-analysis aimed to evaluate the effectiveness of telemedicine interventions in patients with OA and IA, encompassing a broader spectrum of telemedicine modalities beyond exercise-based telerehabilitation. By including both OA and IA populations and considering diverse telemedicine approaches—including remote consultation, mobile health applications, video-based rehabilitation, digital health education, and self-management support—this review sought to provide a comprehensive evidence synthesis that addresses the limitations of previous reviews and clarifies the current state of evidence for telemedicine in arthritis care.

## 2 Methods

This review was registered in PROSPERO (CRD420261401824). The review and reporting processes were undertaken in accordance with the Cochrane Handbook for Systematic Reviews of Interventions and the PRISMA statement (16). In brief, we searched PubMed, Embase, the Cochrane Library, Web of Science, and WHO ICTRP databases from inception to 10 July 2026. Two reviewers independently screened records, extracted data, and assessed risk of bias using the Cochrane RoB 2 tool. Continuous outcomes were pooled using random-effects meta-analysis and reported as standardized mean differences with 95% confidence intervals. The certainty of evidence was evaluated using the GRADE framework.

### 2.1 Search strategy and study selection

We searched PubMed, EMBASE, Web of Science, the Cochrane Library, and the WHO ICTRP from inception to 10 July 2026 for all studies investigating telemedicine interventions in patients with OA or IA. The search strategy combined Medical Subject Headings (MeSH) and free-text terms using Boolean operators, with no restrictions on article type. Supplementary Table S1 provides the full search terms and procedures.

Retrieved records were managed with EndNote X9 software and duplicate entries were removed by automatic detection and manual verification. Two authors independently performed the study selection by screening titles and abstracts, followed by full-text eligibility assessment.

### 2.2 Inclusion and exclusion criteria

The selection criteria were developed based on the PICOS framework. Population (P): patients diagnosed with OA or IA. Intervention (I): telemedicine interventions, defined as healthcare services delivered through information and communication technologies when patients and healthcare providers are geographically separated, including remote consultation and assessment, follow-up management and disease monitoring, health education, behavioral and rehabilitation support, medication management, and health information and self-care support. Comparison (C): standard care for OA or IA, wait list control, or no intervention. Outcomes (O): pain, quality of life, self-efficacy, physical function, and disease activity. Study design (S): randomized controlled trials (RCTs) only.

In our analysis, we did not include studies with one or more of the following characteristics: (1) non-randomized controlled trials; (2) unavailable full text, including conference abstracts and study protocols; (3) interventions inconsistent with the eligibility criteria; (4) absence of relevant outcome measures; and (5) data unavailable for extraction after contacting the corresponding authors. With regard to language restrictions, only studies published in English were eligible for inclusion, and non-English publications were excluded. While this criterion facilitated consistent study screening and data abstraction across the review team, we acknowledge that it carries a potential risk of language bias, as clinically relevant studies conducted in non-English-speaking regions and published in local languages such as Chinese, Spanish, or Portuguese may not have been captured, which could overrepresent positive findings from English-language literature and affect the generalizability of the pooled effect estimates.

### 2.3 Data abstraction

Two reviewers independently recorded the study data in a standardized Excel form. The information collected included author, publication year, country, sample size, intervention and control measures, study duration, participant characteristics (age and gender), outcome measures, and assessment methods. For publications with missing or incomplete data, we requested the missing information from the corresponding authors.

### 2.4 Risk of bias and certainty of evidence assessment

Risk of bias for each eligible randomized controlled trial was assessed independently by two trained reviewers using the revised Cochrane Risk of Bias tool (RoB 2.0), which covers five distinct domains: bias arising from the randomization process (D1), bias due to deviations from the intended interventions (D2), bias due to missing outcome data (D3), bias in measurement of the outcome (D4), and bias in selection of the reported result (D5). Each domain was assigned a judgment of “low risk,” “some concerns,” or “high risk,” and an overall risk-of-bias rating was generated for each study based on the domain-level assessments. Any disagreements between the two reviewers were resolved via discussion with a third senior investigator until consensus was reached (17). After the two reviewers independently completed the RoB 2 risk-of-bias assessments for all studies, interrater agreement was calculated using Cohen’s Kappa; the overall Kappa value for the initial assessments was 0.78, indicating good interrater agreement.

### 2.5 Statistical analysis

For continuous outcomes, means and standard deviations (SDs) were extracted. When data were reported as medians and interquartile ranges, the corresponding means and SDs were estimated using the method of Wan et al. (18). When studies reported only medians and ranges, means and SDs were estimated using the method of Hozo et al. (19). For trials reporting both baseline and endpoint data, change-from-baseline scores and their corresponding SDs were preferentially extracted. When the SD of the change score was unavailable, it was estimated from the baseline and endpoint SDs using the method recommended by Follmann et al. (20), with the correlation coefficient set at r = 0.5 (this value is the standard recommendation from the Cochrane Handbook for Systematic Reviews of Interventions for handling missing variance data regarding continuous outcomes; its impact on the direction, statistical significance, and magnitude of the pooled effect size falls within an acceptable range and does not alter the study’s core conclusions). For multi-arm trials, we extracted data only from the intervention and control groups relevant to the PICO framework of this review and excluded the other arms; when a multi-arm trial included multiple eligible intervention groups, the relevant groups were combined into a single group.

Because most outcomes were continuous variables and measurement scales or scoring methods differed among studies, SMDs with 95% CIs were adopted as the pooled effect estimates. We assessed between-study heterogeneity via the *χ*^2^ test and *I*^2^ statistic, with substantial heterogeneity defined as a *P*-value < 0.10 or an *I*^2^ value exceeding 50%. Considering the inherent differences in disease type, telemedicine modality, intervention duration, and outcome measurement tools among included studies, we applied a random-effects model for all effect estimations, and statistical significance was set at a two-sided *P*-value < 0.05. To comprehensively reflect the impact of heterogeneity on the uncertainty of the results, we calculated 95% prediction intervals for each primary outcome. The prediction intervals were based on a random-effects model—with the between-study variance (*τ*^2^) estimated using the DerSimonian–Laird method—and were calculated as follows: 95%PI=μRE^±t0.975,k−2×SE2(μ^RE)+τ2, where *k* represents the number of included studies and SE represents the standard error of the pooled effect estimate. This method follows the guidelines for calculating prediction intervals in meta-analysis proposed by Borenstein et al. Compared with a normal distribution approximation, prediction intervals based on the *t*-distribution yield more conservative results when the number of included studies is small, making them better suited to the characteristics of this study, where the number of included studies for each outcome is limited (21). All calculations were conducted in Review Manager version 5.4, with *τ*^2^ values extracted directly from the software output. Subgroup analyses of the primary outcomes were conducted according to intervention duration, type of control group, and disease type. We conducted leave-one-out sensitivity analyses for primary outcomes to evaluate the robustness of our pooled estimates, and for outcomes with data from 10 or more studies, we assessed potential publication bias using funnel plots and Egger’s regression test.

## 3 Results

### 3.1 Study selection process and characteristics of included studies

Through systematic literature retrieval, we found 8,211 records from PubMed (*n* = 1,630), Embase (*n* = 2,961), Web of Science (*n* = 1,485), the Cochrane Library (*n* = 1,904), and WHO ICTRP (*n* = 231). Figure 1 presents the study selection process. The key features of the 24 eligible RCTs are summarized in Table 1. Among these studies, 18 focused on osteoarthritis and 6 on inflammatory arthritis, including four studies on rheumatoid arthritis and two on ankylosing spondylitis. Across all included studies, 2,262 participants were evaluated, comprising 1,568 patients with OA and 694 patients with IA. Trials were performed in 13 countries including Turkey (*n* = 4) (22–25); South Korea (26–28) and Iran (29–31)(*n* = 3 each); Saudi Arabia (32, 33), Australia (34, 35), Germany (36, 37), and China (38, 39) (*n* = 2 each); and Brazil (40), the United States (41), the Netherlands (42), Belgium (43), Denmark (44), and Spain (45) (*n* = 1 each). Sample sizes ranged from 25 to 239 participants, and intervention durations ranged from 6 weeks to 2 years. The telemedicine interventions mainly included mobile applications, video- or online-based rehabilitation training, telephone follow-up, digital health education, WeChat-based management, and remote exercise guidance.

**Figure 1:**
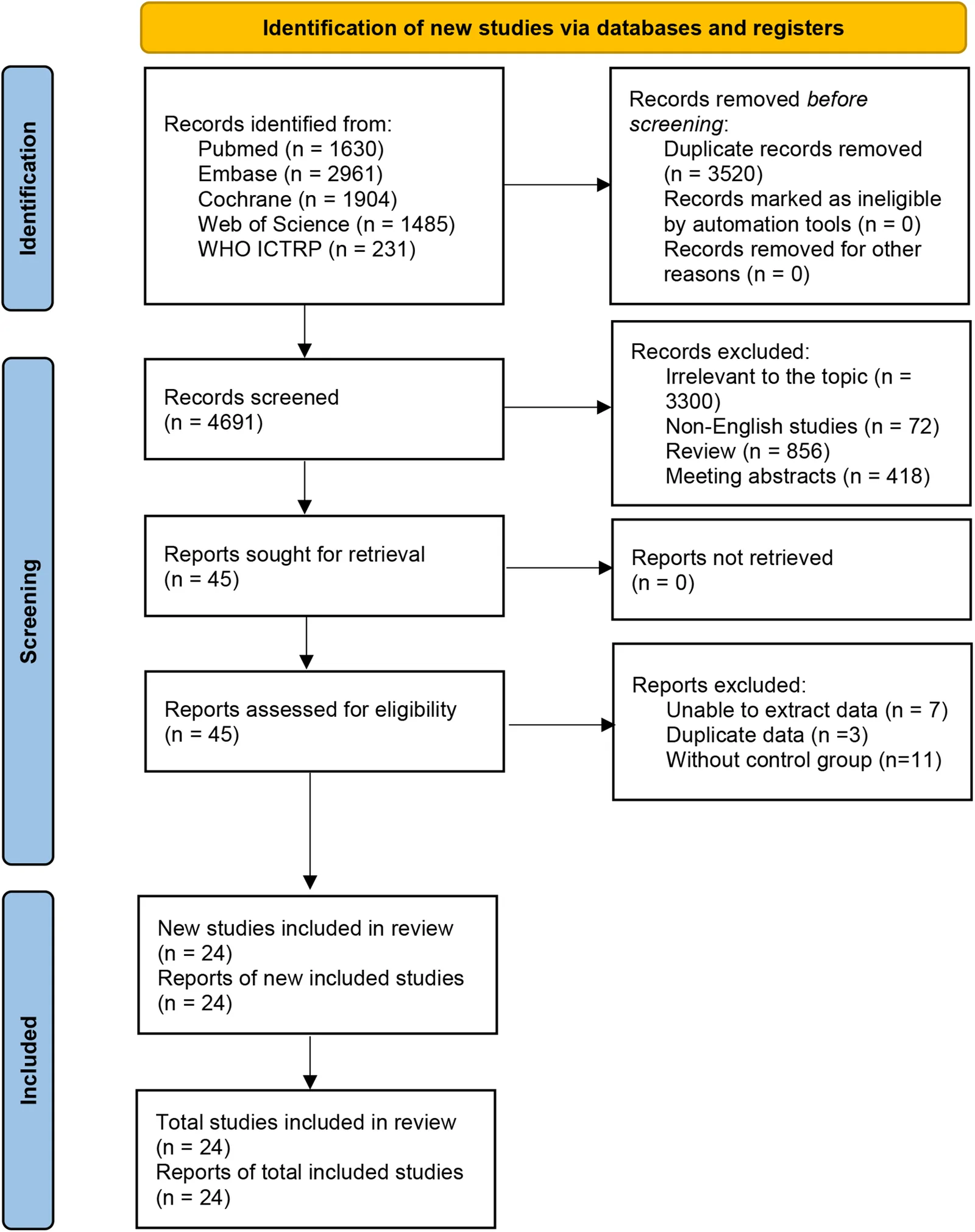
PRISMA flow diagram of study selection.

**Table 1:** Characteristics of the 24 included randomized controlled trials evaluating telemedicine interventions in patients with osteoarthritis and inflammatory arthritis.

Study	Country	Trial registration	Population	Intervention	Control	Intervention duration	Patients, *n* (T/C)	Mean/median age (T/C)	Female, *n* (T/C)
Aily et al. (40)	Brazil	RBR-662hn2	Patients with knee osteoarthritis	Circuit training delivered via telerehabilitation using video recordings, followed by periodic phone calls in order to motivate and instruct participants	Same circuit training program in a face-to-face format	14 weeks	50/50	53/55	30/30
Alasfour and Almarwani (32)	Saudi Arabia	NCT04159883	Female older adults with knee osteoarthritis	Home exercise programs using an Arabic smartphone application called “My Dear Knee”	Home exercise programs as handouts	6 weeks	20/20	53.65/55.15	20/20
Arfaei Chitkar et al. (29)	Iran	IRCT20161208031300N2	Female patients with knee osteoarthritis	Mobile app-based instruction coupled with routine care	Routine care	2 months	31/29	57.84/58.52	31/29
Azma et al. (30)	Iran	IRCT2017013032251N2	Patients with knee osteoarthritis	Home-based telerehabilitation	Office-based physical therapy	6 weeks	27/27	55/56	NA/NA
Baker et al. (41)	USA	NCT01394874	Elderly Patients With Knee Osteoarthritis	Telephone-based, motivational, strength training exercise adherence counseling intervention	Strength train	2 years	52/52	65.8/64.5	42/43
Bennell et al. (34)	Australia	ACTRN12618000930280	Patients with symptomatic knee osteoarthritis	The exercise program comprised six physiotherapist consultations via videoconference for exercise, self-management advice, and behavioral counseling, plus exercise equipment and resources	Electronic osteoarthritis information	6 months	172/67	65.4/65.3	93/45
Ceylan et al. (22)	Turkey	NCT05727488	Individuals with rheumatoid arthritis	Remote video-based Strengthening and Stretching for Rheumatoid Arthritis of the Hand	No additional intervention	12 weeks	36/37	53/52	35/30
Cuperus et al. (42)	Netherlands	NTR2137	Patients with generalized osteoarthritis	Telephone-based treatment program	Face-to-face treatment program	6 weeks	72/75	59/61	61/64
Dieter et al. (36)	Germany	DRKS00030932	Patients With Knee Osteoarthritis	Self-directed mobile health exercise intervention	Usual care	12 weeks	98/96	61.6/62.1	70/62
Doumen et al. (43)	Belgium	NCT05888181	Patients with rheumatoid arthritis	App-based self-management intervention	Routine care	16 weeks	60/62	58/58	38/45
Gunes Gencer et al. (23)	Turkey	NCT05771376	Patients with ankylosing spondylitis	Baduanjin qigong online exercise program	Home exercises	12 weeks	29/30	46.32/49.53	20/21
Hinman et al. (35)	Australia	ACTRN12621000724875	Adults with knee osteoarthritis	Physiotherapist-prescribed strength exercises and the “My Exercise Messages” app	Physiotherapist-prescribed strength exercises	26 weeks	90/92	64/63.1	63/63
Im et al. (26)	Korea	NCT05197010	Older Patients With Chronic Knee Osteoarthritis	Mobile app-based personalized self-exercise program with remote symptom monitoring and weekly exercise adjustment	Initial individualized exercise guidance plus a printed exercise program brochure	6 weeks	19/10	69.9/71.4	16/8
Khazaei et al. (31)	Iran	IRCT20220917055973N1	Patients With Obesity and Knee Osteoarthritis	Tele-educational videos on nutrition, lifestyle changes, physical activity, individualized exercises, and psychosocial support	Oral advice on diet and exercise	8 weeks	14/11	58.8/59.47	12/10
Knudsen et al. (44)	Denmark	NCT04669340	Patients with rheumatoid arthritis	Digital patient education	Face-to-face patient education	12 months	89/91	59/59	54/56
Lee et al. (27)	Korea	KCT0006170	Older Women With Knee Osteoarthritis	Home-based remote rehabilitation lower-extremity strengthening exercises	No intervention	8 weeks	19/16	65.63/68.27	19/16
Nambi et al. (33)	Saudi Arabia	NCT06430242	Overweight and obese elderly women with locomotor disabilities from knee osteoarthritis	Telerehabilitation (supervised training)	Brochure explaining how to do each exercise	8 weeks	28/28	56.1/57.5	28/28
Oh et al. (28)	Korea	KCT0007523	Patients with rheumatoid arthritis	The mHealth intervention programs comprised eight modules, each including videos and booklets.	Elastic TheraBands, therapeutic putty, and booklets for the educational and exercise sessions through an in-person short education session	8 weeks	73/69	52.85/55.54	62/61
Rodríguez et al. (45)	Spain	NCT04263974	People with symptomatic hand osteoarthritis	Home multimodal treatment (exercise, education, and self-management recommendations) with the carehand mobile app	Usual care (written exercises)	12 weeks	34/40	62.2/64.3	25/25
Song et al. (38)	China	ChiCTR-IPR-16009293	Patients with Ankylosing Spondylitis	Four individual educational sessions and educational information sharing through WeChat, the predominant social networking app in China	Standard care	12 weeks	59/59	30.8/29.1	14/11
Tümtürk et al. (24)	Turkey	NCT05209893	Patients With Knee Osteoarthritis	Home-based telerehabilitation exercise and education via WhatsApp	Home-based exercise and education program delivered via printed materials	8 weeks	29/28	53.59/51.5	23/16
Weber et al. (37)	Germany	DRKS00027164	People with hip or knee osteoarthritis	Handout from the study staff with information on how to install the app and instructions on how to get started	Usual care	12 weeks	32/28	60/64	21/16
Yilmaz and Polat (25)	Turkey	NCT04816474	Older Adults With Osteoarthritis	Online training for the first 4 weeks and telephone counseling for the following 4 weeks	No additional intervention	8 weeks	26/26	67.62/68.96	18/19
Zhu et al. (15)	China	NCT06220565	People With Knee Osteoarthritis	CV-based exercise assessment and intervention system designed specifically for patients with knee osteoarthritis	Exercise rehabilitation education video	6 weeks	32/28	68.59/67.64	19/15

T/C, telemedicine/control; NA, not available; CV, computer vision.

### 3.2 Risk of bias

The RoB 2.0 assessment results are visualized in Supplementary Figure S1 and S2. Overall, majority of the included studies were judged to have some concerns in terms of overall risk of bias, and only one study (Ceylan 2025) was classified as having high overall risk of bias, which was driven by a high-risk rating for deviations from the intended interventions. At the domain level, most studies exhibited low risk of bias for the randomization process, missing outcome data, and selection of the reported result. By contrast, most studies raised some concerns regarding outcome measurement, as the nature of telemedicine interventions made it infeasible to blind participants and intervention providers to group allocation. No study was rated at high risk for the randomization process, missing outcome data, outcome measurement, or selective reporting domains.

### 3.3 Meta-analysis results

#### 3.3.1 Pain scores

Twelve studies reported pain scores, including 527 individuals in the telemedicine group and 419 in the control group, as presented in Figure 2a. Random-effects meta-analysis showed that telemedicine interventions reduced pain scores (SMD = −0.66, 95% CI: −1.00 to −0.32, *P* = 0.0001); however, the 95% prediction interval (−1.90 to 0.58) included the null value. Substantial between-study heterogeneity was observed (*I*^2^ = 82%, *P* < 0.00001).

**Figure 2:**
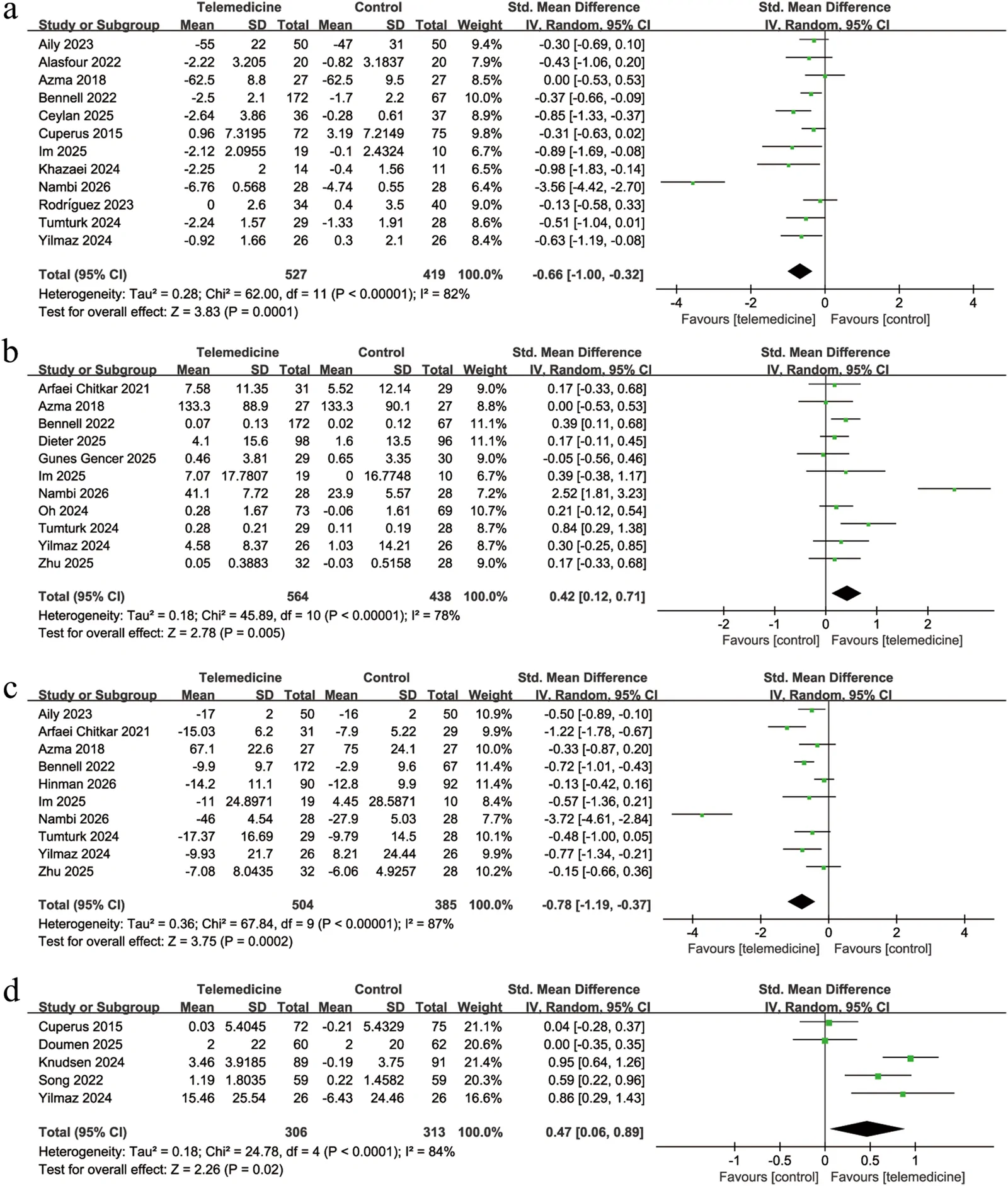
Forest plots showing the effects of telemedicine interventions compared with control conditions on pain scores, quality of life, WOMAC scores, and self-efficacy in patients with arthritis: **(a)** pain scores; **(b)** quality of life; **(c)** WOMAC scores; and **(d)** self-efficacy.

#### 3.3.2 Quality of life

Eleven studies reported outcomes related to quality of life, including 564 individuals in the telemedicine group and 438 in the control group, as presented in Figure 2b. Random-effects meta-analysis indicated that telemedicine interventions were associated with improved quality of life (SMD = 0.42, 95% CI: 0.12 to 0.71, *P* = 0.005); however, the 95% prediction interval (−0.60 to 1.44) included the null value. Substantial between-study heterogeneity was observed (*I*^2^ = 78%, *P* < 0.00001).

#### 3.3.3 McMaster universities osteoarthritis index

Ten studies reported Western Ontario and McMaster Universities Osteoarthritis Index (WOMAC) scores, including 504 individuals in the intervention group and 385 in the control group, as presented in Figure 2c. Random-effects meta-analysis demonstrated reduced WOMAC scores with telemedicine interventions (SMD = −0.78, 95% CI: −1.19 to −0.37, *P* = 0.0002); however, the 95% prediction interval (−2.25 to 0.69) included the null value. Substantial between-study heterogeneity was detected (*I*^2^ = 87%, *P* < 0.00001).

#### 3.3.4 Self-efficacy

Five studies evaluated outcomes associated with self-efficacy, including 306 individuals in the telemedicine group and 313 in the control group, as presented in Figure 2d. Random-effects meta-analysis indicated that telemedicine interventions were associated with improved self-efficacy (SMD = 0.47, 95% CI: 0.06 to 0.89, *P* = 0.02); however, the 95% prediction interval (−1.04 to 1.98) included the null value. Substantial between-study heterogeneity was detected (*I*^2^ = 84%, *P* < 0.0001).

#### 3.3.5 Secondary outcomes

Three included trials (27, 37, 41) contributed only to secondary outcomes and are therefore presented in Supplementary Figure S3 rather than in the main forest plots. Additional studies reported other functional and disease activity-related outcomes, including the Knee injury and Osteoarthritis Outcome Score (KOOS), Timed Up and Go Test (TUG), Rheumatoid Arthritis Disease Activity Index (RADAI), and Pain Catastrophizing Scale (PCS). Due to the small evidence base for each outcome, random-effects models were adopted in the meta-analysis.

Five studies reported TUG scores, including 133 individuals assigned to the telemedicine group and 117 to the control group. Telemedicine interventions significantly reduced TUG scores (SMD = −0.44, 95% CI: −0.80 to −0.09, *P* = 0.01). Moderate heterogeneity was observed, but it was not significant (*I*^2^ = 41%, *P* = 0.15). However, the telemedicine and control groups did not differ significantly in KOOS, RADAI, or PCS scores. Detailed results are presented in Supplementary Figure S3.

#### 3.3.6 Subgroup analysis by intervention duration

Subgroup analyses were performed for pain scores, quality of life, and WOMAC scores according to intervention duration, with studies categorized into <8, 8, and >8 weeks subgroups.

For pain scores, telemedicine interventions reduced pain in all three subgroups: <8 weeks (SMD = −0.32, 95% CI: −0.59 to −0.05, *P* = 0.02), 8 weeks (SMD = −1.39, 95% CI: −2.60 to −0.18, *P* = 0.02), and >8 weeks (SMD = −0.40, 95% CI: −0.65 to −0.14, *P* = 0.002). Compared with overall heterogeneity (*I*^2^ = 82%), heterogeneity decreased to low or moderate levels in the <8- and >8-week subgroups (*I*^2^ = 12% and 40%, respectively), whereas substantial heterogeneity remained in the 8-week subgroup (*I*^2^ = 92%). Subgroup differences were not significant (*P* = 0.23), as presented in Figure 3a.

**Figure 3:**
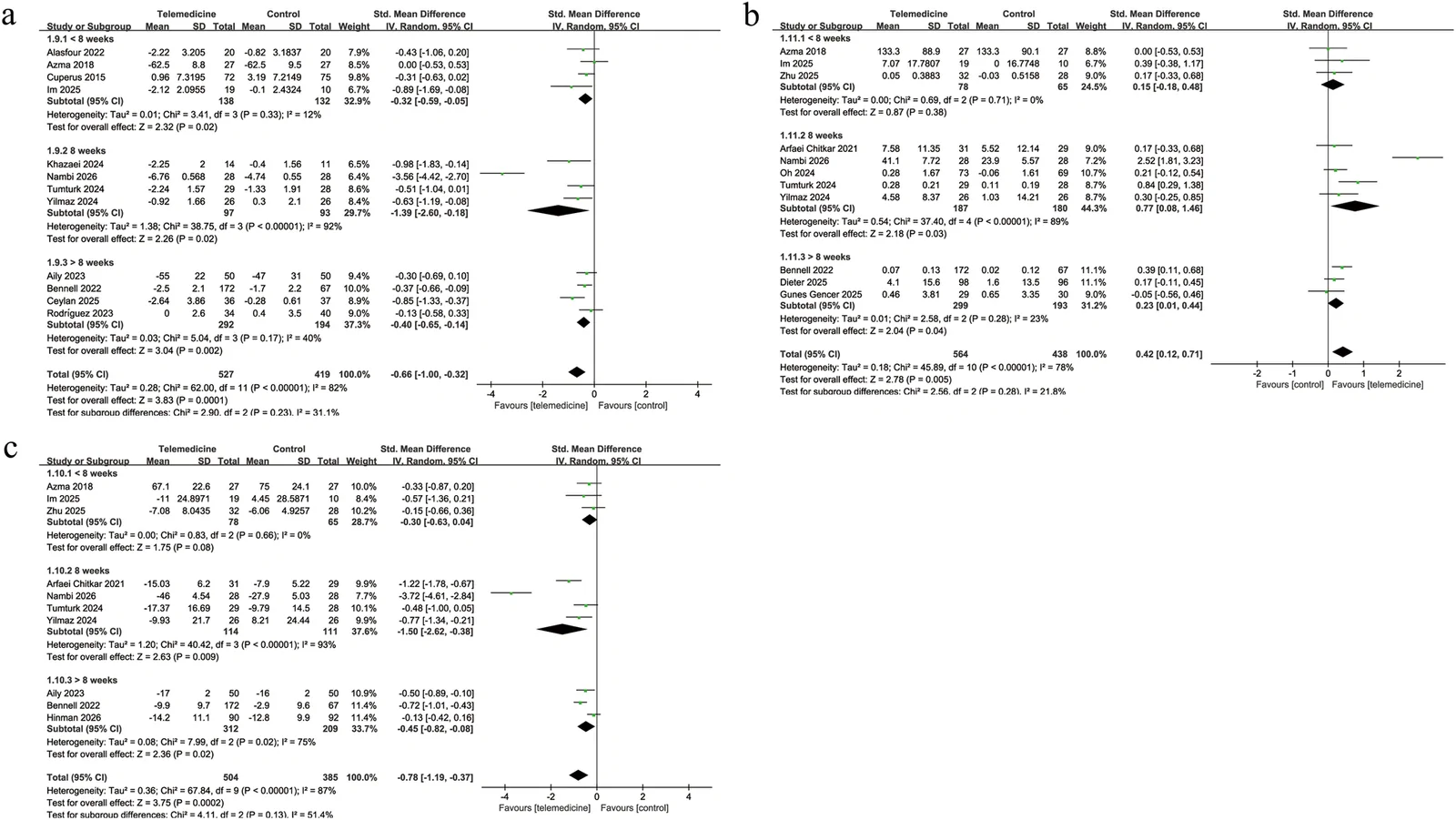
Subgroup analyses of the effects of telemedicine interventions on pain scores, quality of life, and WOMAC scores stratified by intervention duration (<8, 8, and >8 weeks): **(a)** pain scores; **(b)** quality of life; and **(c)** WOMAC scores.

For quality of life, interventions lasting <8 weeks did not show an improvement (SMD = 0.15, 95% CI: −0.18 to 0.48, *P* = 0.38), whereas interventions lasting 8 and >8 weeks improved quality of life, with SMDs of 0.77 (95% CI: 0.08 to 1.46, *P* = 0.03) and 0.23 (95% CI: 0.01 to 0.44, *P* = 0.04), respectively. The overall heterogeneity was 78%; however, heterogeneity decreased markedly in the <8- and >8-week subgroups (*I*^2^ = 0% and 23%, respectively), but remained substantial in the 8-week subgroup (*I*^2^ = 89%). Between-subgroup differences were not considered statistically significant (*P* = 0.28), as presented in Figure 3b.

For WOMAC scores, interventions lasting <8 weeks did not demonstrate an effect (SMD = −0.30, 95% CI: −0.63 to 0.04, *P* = 0.08), whereas interventions lasting 8 and >8 weeks reduced WOMAC scores, with SMDs of −1.50 (95% CI: −2.62 to −0.38, *P* = 0.009) and −0.45 (95% CI: −0.82 to −0.08, *P* = 0.02), respectively. Compared with overall heterogeneity (*I*^2^ = 87%), heterogeneity decreased in the <8-week subgroup (*I*^2^ = 0%), while substantial heterogeneity remained in the 8- and >8-week subgroups (*I*^2^ = 93% and 75%, respectively). No statistically significant differences between subgroups were identified (*P* = 0.13), as presented in Figure 3c. It should be noted that the absence of a statistically significant difference between subgroups does not imply that the effects across subgroups are equivalent. Due to limitations regarding the number of included studies and the total sample size, subgroup analyses in this study lacked sufficient statistical power to detect actual differences between groups; consequently, all subgroup analyses are exploratory in nature and cannot be used to guide clinical decision-making.

#### 3.3.7 Subgroup analysis by control group type

Subgroup analyses of pain scores, quality of life, and WOMAC scores were conducted according to control group type, with studies classified as using either usual care/no intervention or an active comparator.

According to subgroup analyses, telemedicine resulted in lower pain scores than usual care/no intervention (SMD = −0.53, 95% CI: −0.97 to −0.08, *P* = 0.02) or active comparator (SMD = −0.73, 95% CI: −1.17 to −0.29, *P* = 0.001). Heterogeneity was moderate and substantial in the two subgroups, respectively (*I*^2^ = 58% and 86%). No statistically significant difference was observed between the subgroups (*P* = 0.53), as presented in Figure 4a.

**Figure 4:**
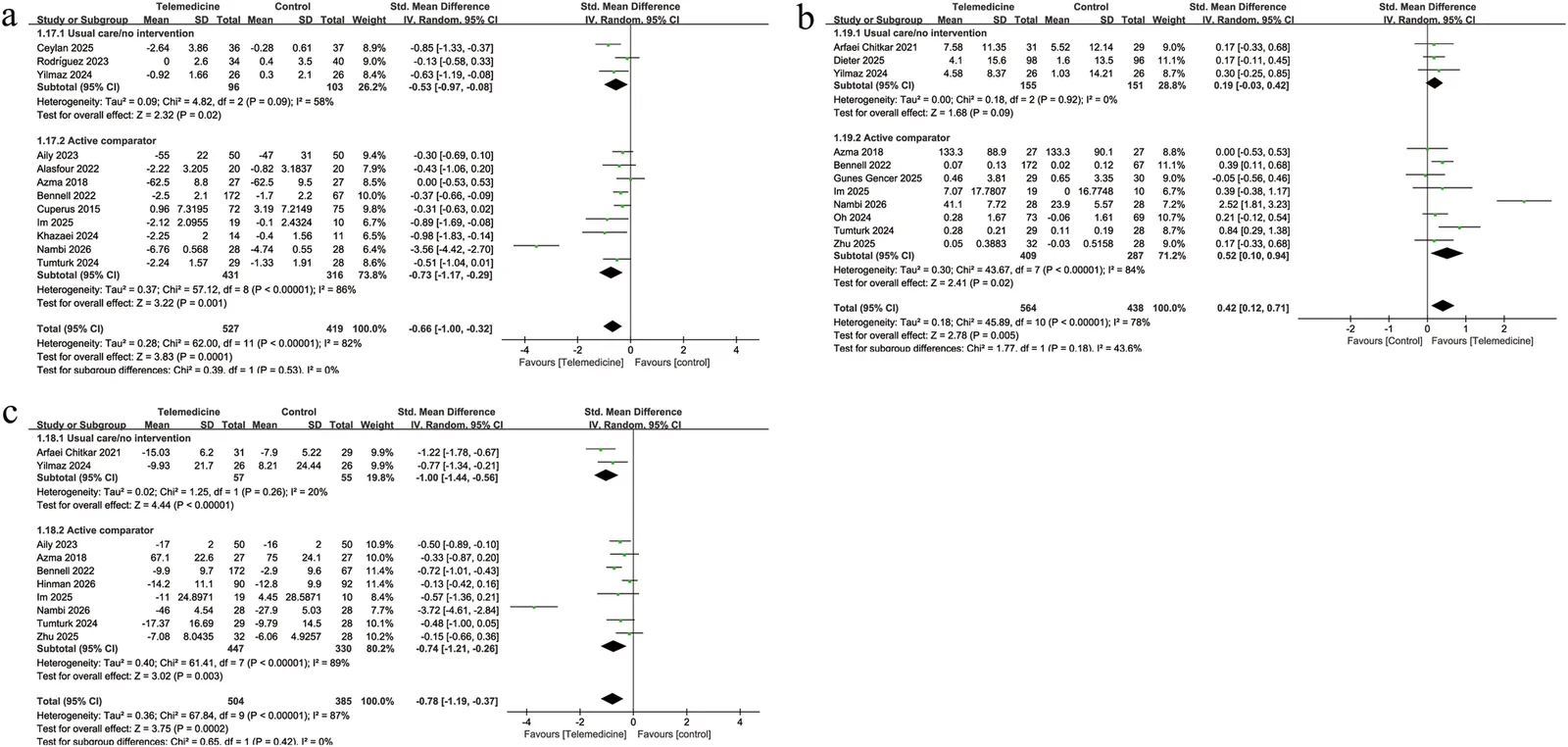
Subgroup analyses of the effects of telemedicine interventions on pain scores, quality of life, and WOMAC scores stratified by control group type (usual care/no intervention vs. active comparator): **(a)** pain scores; **(b)** quality of life; and **(c)** WOMAC scores.

According to subgroup analyses, telemedicine was not associated with improved quality of life compared with usual care/no intervention (SMD = 0.19, 95% CI: −0.03 to 0.42, *P* = 0.09), but was associated with better quality of life than the active comparator (SMD = 0.52, 95% CI: 0.10 to 0.94, *P* = 0.02). No heterogeneity was detected in the usual care/no intervention subgroup (*I*^2^ = 0%), whereas substantial heterogeneity was present in the active comparator subgroup (*I*^2^ = 84%). No statistically significant differences were observed between the subgroups (*P* = 0.18), as presented in Figure 4b.

Subgroup analyses demonstrated that telemedicine was associated with lower WOMAC scores than usual care/no intervention (SMD = −1.00, 95% CI: −1.44 to −0.56, *P* < 0.00001) and active comparator (SMD = −0.74, 95% CI: −1.21 to −0.26, *P* = 0.003). Heterogeneity was low and substantial in the two subgroups, respectively (*I*^2^ = 20% and 89%). No significant subgroup differences were identified (*P* = 0.42), as presented in Figure 4c. Due to limitations regarding the number of included studies and the total sample size, the subgroup analyses in this study lacked sufficient statistical power to detect actual differences between groups; consequently, all subgroup analyses are exploratory in nature and cannot be used to guide clinical decision-making.

#### 3.3.8 Subgroup analysis by disease type

Subgroup analyses of pain, quality of life, and self-efficacy were performed according to disease type, with eligible studies categorized into OA and IA subgroups. It should be noted that, given the inclusion of only two to three studies and the limited total sample size for the IA subgroup, the absence of a statistically significant difference between subgroups does not imply that the effects of telemedicine are comparable across the two disease categories; rather, it indicates only that current evidence has not detected a significant intergroup difference, and equivalence of effect cannot be inferred from this finding.

In the OA subgroup, which included 11 studies, telemedicine interventions were associated with reduced pain scores (SMD = −0.70, 95% CI: −1.13 to −0.28, *P* = 0.001), although substantial between-study heterogeneity was observed (*I*^2^ = 85%). Only one study was included in the IA subgroup, and its findings also indicated an association between telemedicine and reduced pain scores (SMD = −0.85, 95% CI: −1.33 to −0.37, *P* = 0.0005); consequently, between-study heterogeneity could not be evaluated. The differences between the OA and IA subgroups were not statistically significant (*P* = 0.65), as presented in Supplementary Figure S4a.

In the OA subgroup, which included nine studies, telemedicine interventions were associated with better quality of life (SMD = 0.51, 95% CI: 0.14 to 0.87, *P* = 0.006), although substantial between-study heterogeneity was identified (*I*^2^ = 81%). Two studies were included in the IA subgroup, in which quality of life did not differ significantly between the telemedicine and control groups (SMD = 0.13, 95% CI: −0.15 to 0.41, *P* = 0.36), with no observed heterogeneity (*I*^2^ = 0%). No significant subgroup differences were detected between the OA and IA subgroups (*P* = 0.11), as presented in Supplementary Figure S4b.

In the OA subgroup, which included two studies, self-efficacy did not differ significantly between the telemedicine and control groups (SMD = 0.42, 95% CI: −0.38 to 1.22, *P* = 0.31), and substantial between-study heterogeneity was identified (*I*^2^ = 83%). In the IA subgroup, which included three studies, self-efficacy was also comparable between the telemedicine and control groups (SMD = 0.52, 95% CI: −0.04 to 1.07, *P* = 0.07), with substantial between-study heterogeneity (*I*^2^ = 87%). No significant subgroup differences were detected between the OA and IA subgroups (*P* = 0.84), as presented in Supplementary Figure S4c. Due to limitations regarding the number of included studies and the total sample size, subgroup analyses in this study lacked sufficient statistical power to detect actual differences between groups; consequently, all subgroup analyses are exploratory in nature and cannot be used to guide clinical decision-making.

### 3.4 Publication bias

For outcomes reported in at least 10 studies, publication bias was assessed, including pain scores, quality of life, and WOMAC scores. Funnel plots showed no obvious asymmetry for these outcomes, as presented in Supplementary Figure S5. According to Egger’s regression test, publication bias was not statistically significant for pain scores, quality of life, or WOMAC scores (*P* = 0.076, *P* = 0.148, and *P* = 0.194, respectively). Detailed results are presented in Supplementary Table S2. However, the negative intercept from Egger’s regression test for pain suggested that smaller studies may have reported more favorable intervention effects, and the possibility of small-study effects or publication bias could therefore not be entirely ruled out. Accordingly, publication bias for pain was considered strongly suspected in the GRADE assessment.

### 3.5 Sensitivity analysis

Sensitivity analysis using the leave-one-out method was performed to verify the robustness of the findings. The pooled estimates for pain scores, quality of life, and WOMAC scores remained consistent after omitting each study one at a time, with statistical significance preserved. In particular, after excluding Nambi et al. (33), the pooled SMD for pain was −0.41 (95% CI: −0.56 to −0.26, *P* < 0.00001), with low heterogeneity (*I*^2^ = 14%). The pooled SMD for quality of life was 0.25 (95% CI: 0.12 to 0.38, *P* = 0.0002), with no observed heterogeneity (*I*^2^ = 0%). For WOMAC scores, the pooled SMD was −0.52 (95% CI: −0.75 to −0.29, *P* < 0.00001), although substantial heterogeneity remained (*I*^2^ = 56%). For self-efficacy, sensitivity analysis showed that after excluding some studies, the 95% CI of the pooled effect crossed the line of no effect, suggesting relatively limited stability for this outcome. Overall, the main outcome findings of this study were generally robust. Detailed results are presented in Supplementary Figure S6. A sensitivity analysis forest plot, after excluding Nambi et al. (33), is shown in Supplementary Figure S7.

### 3.6 GRADE assessment of the certainty of evidence

A systematic review using the GRADE framework was conducted to assess the certainty of evidence for the four primary outcomes of this study. The results indicated that the certainty of evidence for all primary outcomes ranged from very low to low. In particular, the certainty of evidence for pain scores (based on 12 randomized controlled trials) was very low, downgraded due to serious risk of bias, serious inconsistency (indicated by 82% interstudy heterogeneity), and highly suspected publication bias (suggested by Egger’s test). The certainty of evidence for quality of life (based on 11 studies) was also very low, downgraded due to serious risk of bias, serious inconsistency, and serious imprecision (resulting from wide confidence intervals for effect sizes and limited total sample size). The certainty of evidence for WOMAC scores (based on 10 studies) was low, downgraded due to serious risk of bias and serious inconsistency. The certainty of evidence for self-efficacy (based on only five studies) was very low, downgraded due to serious risk of bias, serious inconsistency, and serious imprecision; no assessment for publication bias was performed due to the insufficient number of included studies. No serious issues regarding indirectness were found for any of the outcomes, nor were any factors identified—such as plausible confounding, effect size magnitude, or dose–response gradients—that would warrant upgrading the certainty of evidence. Detailed results are presented in Supplementary Table S3.

## 4 Discussion

This review synthesized evidence from 24 RCTs to examine how telemedicine affects health-related outcomes in patients with OA and IA. The findings suggested that telemedicine was associated with improvements in pain scores, quality of life, self-efficacy, and some functional outcomes in patients with arthritis. However, considerable heterogeneity across studies and wide prediction intervals for pain scores, quality of life, self-efficacy, and WOMAC scores indicated substantial uncertainty regarding these effects. Evidence for self-efficacy was derived from only five trials, and leave-one-out sensitivity analyses caused the 95% CI to include the null value after removal of some studies, suggesting that the pooled estimate was sensitive to individual trials. The GRADE assessment classified the certainty of evidence as very low for pain scores, quality of life, and self-efficacy and low for WOMAC scores. Overall, in this study, interstudy heterogeneity for the four primary outcomes—pain, quality of life, WOMAC scores, and self-efficacy—exceeded 75%, indicating a high level of heterogeneity. This characteristic carries significant clinical implications: The pooled effect size represents only the average effect across the included studies and cannot be directly equated to the actual efficacy of a specific telehealth model, patient population, or clinical setting. Consequently, the pooled value should not be applied indiscriminately; instead, clinical application requires a comprehensive assessment that considers factors such as the intensity and format of the intervention, the severity of the patient’s condition and their self-management capabilities, and the nature of the control intervention. Furthermore, the 95% prediction intervals for all primary outcomes included zero, suggesting that, in real-world clinical practice, similar telehealth interventions could yield null or even negative results. This implies that the statistical significance of the pooled effect alone is insufficient to conclude that telehealth inevitably provides benefits; its clinical value requires further validation through studies with larger sample sizes and more homogeneous designs.

Pain is the most common manifestation of arthritis and is the main priority in its clinical management. Regular tele-exercise or remotely supervised rehabilitation may strengthen the quadriceps and hip musculature; improve joint stability, proprioception, and range of motion; and consequently alleviate pain and functional impairment (46). In patients with overweight or obesity, exercise may support weight management, decrease mechanical joint loading, and further relieve mechanically mediated pain (47). Moreover, ongoing feedback, reminders, and monitoring delivered through remote platforms may enhance exercise adherence and help patients integrate rehabilitation into sustained self-management rather than treating it as a time-limited intervention. Self-efficacy is associated with pain severity, function impairment, and psychological wellbeing in patients with arthritis (48). Nambi et al. reported significantly larger effect sizes on pain scores, quality of life, and WOMAC scores than in the other studies (33). Only 56 overweight or obese women aged 45–65 years with mild-to moderate knee OA had been enrolled in the trial. Participants in the intervention group completed therapist-supervised, moderate-intensity remote training five times weekly for 8 weeks, whereas those in the control group mainly followed a manual-based self-training program. The unusually large effects may be partly attributable to the selected participant population, intensive training schedule, and unequal levels of supervision between groups; however, this explanation remains speculative. Exclusion of this study reduced the pooled effect estimates for pain scores, quality of life, and WOMAC scores, while leaving their direction and statistical significance unchanged. This suggests that the study affected the magnitude of the pooled effects but did not materially alter the overall conclusions. Given the limited sample size, the small within-group variability in certain post-intervention measures, and the investigators’ suggestion that a familiarization effect arising from repeated measurements may have partly accounted for the marked reduction in VAS scores, the effect estimates and the generalizability of these findings should be viewed cautiously.

When analyses were stratified by intervention duration, heterogeneity was reduced for some outcomes in the <8- and >8-week subgroups, suggesting that intervention duration may account for part of the observed between-study variability. However, the between-subgroup differences were not statistically significant for pain scores, quality of life, or WOMAC scores. The 8-week subgroup retained substantial heterogeneity, and its effect estimates may have been disproportionately influenced by individual studies, particularly Nambi et al. Accordingly, the current evidence does not support the superiority of any specific intervention duration, and these findings should be interpreted as exploratory. Stratification by control group type likewise revealed no significant subgroup differences for pain scores, quality of life, or WOMAC scores. However, the “active comparator” subgroup combined heterogeneous comparison conditions, including non-personally delivered matched-content controls, in-person interventions, and add-on designs. Differences in comparator content, intensity, and mode of delivery may have influenced the observed effect estimates. Accordingly, the findings of this subgroup analysis should be interpreted cautiously. Disease-specific analyses showed no significant differences between the OA and IA subgroups for pain scores, quality of life, or self-efficacy. However, IA and OA differ fundamentally in their pathophysiological mechanisms, treatment goals, patterns of disease activity fluctuation, and requirements for immunosuppressant management; existing research findings cannot be generalized to the entire population of patients with inflammatory arthritis, nor can a definitive conclusion be drawn regarding the universal effectiveness of telemedicine for IA.

The telehealth interventions included in this study encompassed a variety of modalities, such as mobile health applications, video-guided telerehabilitation, telephone-based follow-up management, digital health education, and social platform-based disease management. These interventions differed significantly in terms of the intensity of professional supervision, the frequency of clinician–patient interaction, the complexity of intervention content, and the level of patient autonomy required. In particular, telerehabilitation interventions involving real-time therapist supervision typically offered higher intervention intensity and greater scope for personalization, whereas autonomous mobile applications and digital health education primarily relied on the delivery of standardized content and entailed relatively lower intervention intensity. This heterogeneity in intervention types and implementation models is likely a key clinical driver of the high heterogeneity observed across studies regarding primary outcomes. Due to the limited number of studies available for each intervention modality, we were unable to conduct subgroup analyses in the present study to clarify how intervention type modifies effect sizes. Future research should undertake head-to-head comparisons of the clinical benefits and cost-effectiveness of different telehealth modalities to provide an evidence base for matching appropriate telehealth solutions to patients based on their disease stage and health needs.

Compared with previous reviews (15), the present study evaluated a broader population that included patients with both osteoarthritis and inflammatory arthritis and considered a wider range of telemedicine interventions beyond remotely delivered exercise rehabilitation, thereby extending the evidence base. Nevertheless, this broader scope introduced greater clinical heterogeneity; therefore, the findings do not establish that telemedicine yields comparable effects across different disease populations or models of care delivery.

This study has several limitations. First, the nature of telemedicine made complete blinding of participants and intervention providers difficult; therefore, performance bias cannot be ruled out, and all primary outcomes were downgraded for serious risk of bias in the GRADE assessment. Second, both osteoarthritis and inflammatory arthritis are global conditions, with relevant research published in languages ​​such as Chinese, Spanish, and Portuguese; excluding non-English literature may bias results toward English-language studies—which tend to report more positive findings—thereby overestimating the effect size of telemedicine. Third, marked variation in intervention content and frequency, level of interaction, comparator conditions, intervention duration, and outcome measures contributed to clinical heterogeneity. The resulting substantial heterogeneity and wide prediction intervals limited both the interpretation and generalizability of the pooled estimates. Fourth, the limited number of included studies was insufficient to support a meaningful meta-regression analysis. Consequently, the categorical subgroup analyses had limited statistical power and remained susceptible to study-level confounding. Fifth, evidence for IA was derived from only a small number of studies involving RA and AS, limiting the applicability of the findings to other inflammatory arthritis. Sixth, small sample sizes, imbalances in group allocation, and extreme effect estimates in some trials further increased uncertainty in the pooled findings. Seventh, the absence of 72 non-English records may have contributed language bias and reduced the scope of the evidence. Eighth, the trial was registered after its actual start date, indicating a potential risk of bias associated with retrospective registration. Finally, although Egger’s test was not statistically significant, the pain outcome yielded a *P*-value of 0.076 and a negative intercept. Small-study effects or publication bias therefore remains possible, and the pooled effect for pain may be overestimated. Further high-quality randomized controlled trials, particularly among patients with IA, are needed to investigate the long-term efficacy, safety, cost-effectiveness, and impact of telemedicine on healthcare resource utilization.

## 5 Conclusion

This study indicated that telemedicine was associated with improvements in pain, quality of life, self-efficacy, and selected functional outcomes among patients with arthritis. Although telemedicine was associated with improved self-efficacy, sensitivity analyses suggested that pooled findings were not robust. Evidence certainty was rated as very low for pain scores, quality of life, and self-efficacy and low for WOMAC scores. The 95% prediction intervals for pain scores, quality of life, self-efficacy, and WOMAC scores included the null value, suggesting limited generalizability of the observed effects. Subgroup differences by intervention duration, comparator type, and disease type were not statistically significant. Accordingly, the available evidence neither supports the superiority of any particular intervention duration nor permits firm conclusions about the comparative effectiveness of telemedicine versus any specific comparator strategy. Evidence for IA was limited to a small number of studies in RA and AS, precluding firm conclusions or broad generalization to the wider IA population. Future RCTs are needed with more rigorous designs, larger sample sizes, and longer intervention durations to better clarify the role of telemedicine in the long-term management of arthritis.

## Data Availability

The original contributions presented in the study are included in the article/Supplementary Material, further inquiries can be directed to the corresponding author.

## References

[1] Hunter DJ Bierma-Zeinstra S. Osteoarthritis. Lancet. (2019) 393(10182):1745–59. 10.1016/S0140-6736(19)30417-9 31034380

[2] Ledingham J Snowden N Ide Z. Diagnosis and early management of inflammatory arthritis. Br Med J. (2017) 358:j3248. 10.1136/bmj.j3248 28751303

[3] Steinmetz JD Culbreth GT Haile LM Rafferty Q Lo J Fukutaki KG. Global, regional, and national burden of osteoarthritis, 1990–2020 and projections to 2050: a systematic analysis for the Global Burden of Disease Study 2021. Lancet Rheumatol. (2023) 5(9):e508–22. 10.1016/S2665-9913(23)00163-7 37675071 PMC10477960

[4] Black RJ Cross M Haile LM Culbreth GT Steinmetz JD Hagins H. Global, regional, and national burden of rheumatoid arthritis, 1990–2020, and projections to 2050: a systematic analysis of the Global Burden of Disease Study 2021. Lancet Rheumatol. (2023) 5(10):e594–610. 10.1016/S2665-9913(23)00211-4 37795020 PMC10546867

[5] Kolasinski SL Neogi T Hochberg MC Oatis C Guyatt G Block J. 2019 American College of Rheumatology/Arthritis Foundation Guideline for the management of osteoarthritis of the hand, hip, and knee. Arthritis Care Res (Hoboken). (2020) 72(2):149–62. 10.1002/acr.24131 PMC11488261 31908149

[6] Combe B Landewe R Daien CI Hua C Aletaha D Álvaro-Gracia JM. 2016 Update of the EULAR recommendations for the management of early arthritis. Ann Rheum Dis. (2017) 76(6):948–59. 10.1136/annrheumdis-2016-210602 27979873

[7] Nikiphorou E Santos EJF Marques A Böhm P Bijlsma JWJ Daien CI. 2021 EULAR recommendations for the implementation of self-management strategies in patients with inflammatory arthritis. Ann Rheum Dis. (2021) 80(10):1278–85. 10.1136/annrheumdis-2021-220249 33962964 PMC8458093

[8] Tuckson RV Edmunds M Hodgkins ML. Telehealth. N Engl J Med. (2017) 377(16):1585–92. 10.1056/NEJMsr1503323 29045204

[9] Zhai X Zhou Z Wang Z Liu G Coyte PC. Association between telemedicine adoption and physician job satisfaction: cross-sectional study. J Med Internet Res. (2026) 28:e82285. 10.2196/82285 41915895 PMC13038179

[10] Omboni S Campolo L Panzeri E. Telehealth in chronic disease management and the role of the Internet-of-Medical-Things: the Tholomeus® experience. Expert Rev Med Devices. (2020) 17(7):659–70. 10.1080/17434440.2020.1782734 32536214

[11] Wang H-N Luo P Liu S Liu Y Zhang X Li J. Effectiveness of internet-based telehealth programs in patients with hip or knee osteoarthritis: systematic review and meta-analysis. J Med Internet Res. (2024) 26:e55576. 10.2196/55576 39348685 PMC11474128

[12] Xiang X-N Wang Z-Z Hu J Zhang J-Y Li K Chen Q-X. Telehealth-supported exercise or physical activity programs for knee osteoarthritis: systematic review and meta-analysis. J Med Internet Res. (2024) 26:e54876. 10.2196/54876 39094114 PMC11329855

[13] Venuturupalli S Peck A Jinka Y Fortune N Davuluri N Nowell WB. Home-based telemedicine in rheumatology-A scoping review. ACR Open Rheumatol. (2024) 6(5):312–20. 10.1002/acr2.11660 38456334 PMC11089445

[14] Wu B Zhang M Hu H Wu X. The effectiveness of telemedicine in patients with rheumatoid arthritis: an overview of systematic reviews and meta-analysis. Telemed J E Health. (2023) 29(12):1747–58. 10.1089/tmj.2023.0074 37074335

[15] Zhu J Jiang YS Wang H. Effectiveness of telemedicine-delivered exercise interventions in older adult patients with osteoarthritis: a systematic review and meta-analysis. Front Public Health. (2025) 13:1719841. 10.3389/fpubh.2025.1719841 41415257 PMC12708276

[16] Page MJ McKenzie JE Bossuyt PM Boutron I Hoffmann TC Mulrow CD. The PRISMA 2020 statement: an updated guideline for reporting systematic reviews. Br Med J. (2021) 372:n71. 10.1136/bmj.n71 PMC8005924 33782057

[17] Eldridge S Campbell M Campbell M Dahota A Giraudeau B Higgins J. Revised cochrane risk of bias tool for randomized trials (RoB 2.0): additional considerations for cluster-randomized trials. Cochrane Methods Cochrane Database Syst Rev. (2016) 10(suppl 1):1–17.

[18] Wan X Wang W Liu J Tong T. Estimating the sample mean and standard deviation from the sample size, median, range and/or interquartile range. BMC Med Res Methodol. (2014) 14:135. 10.1186/1471-2288-14-135 25524443 PMC4383202

[19] Hozo SP Djulbegovic B Hozo I. Estimating the mean and variance from the median, range, and the size of a sample. BMC Med Res Methodol. (2005) 5:13. 10.1186/1471-2288-5-13 15840177 PMC1097734

[20] Follmann D Elliott P Suh I Cutler J. Variance imputation for overviews of clinical trials with continuous response. J Clin Epidemiol. (1992) 45(7):769–73. 10.1016/0895-4356(92)90054-Q 1619456

[21] Borenstein M. How to understand and report heterogeneity in a meta-analysis: the difference between I-squared and prediction intervals. Integrative Medicine Research. (2023) 12(4):101014. 10.1016/j.imr.2023.101014 38938910 PMC11208730

[22] Ceylan A Baskan E Inanc I Sarac DC Ozkan A Karaca NB. Effects of the remote video-based SARAH program in individuals with rheumatoid arthritis: a randomized controlled single-blinded study. J Telemed Telecare. (2025) 1357633x251372681. 10.1177/1357633X251372681 40920335

[23] Gunes Gencer GY Cetin SY Kara DS Yardim S Ayan A. The effects of baduanjin qigong exercise via telerehabilitation in ankylosing spondylitis: a randomized controlled study. Explore (NY). (2025) 21(2):103078. 10.1016/j.explore.2024.103078 39580253

[24] Tümtürk İ Bakırhan S Özden F Gültaç E Kılınç CY. Effect of telerehabilitation-based exercise and education on pain, function, strength, proprioception, and psychosocial parameters in patients with knee osteoarthritis: a randomized controlled clinical trial. Am J Phys Med Rehabil. (2024) 103(3):222–32. 10.1097/PHM.0000000000002335 37678215

[25] Yilmaz E Polat Ü. Online disease management training for older adults with osteoarthritis: a randomized controlled trial. Musculoskeletal Care. (2024) 22(3):e1931. 10.1002/msc.1931 39134868

[26] Im YJ Choi J Choi SA Kwon JY Do JG. Feasibility and preliminary effectiveness of a mobile app-based personalized exercise program in older patients with chronic knee osteoarthritis: pilot randomized controlled irial. JMIR Mhealth Uhealth. (2025) 13: e71073. 10.2196/71073 41401375 PMC12707693

[27] Lee E-L Jang MH Lee B-J Han SH Lee HM Choi SU. Home-based remote rehabilitation leads to superior outcomes for older women with knee osteoarthritis: a randomized controlled trial. J Am Med Dir Assoc. (2023) 24(10):1555–61. 10.1016/j.jamda.2023.08.013 37699531

[28] Oh H Suh C-H Kim J-W Boo S. mHealth-based self-management program for patients with rheumatoid arthritis: a pilot randomized controlled study. Nurs Health Sci. (2024) 26(4):e13187. 10.1111/nhs.13187 39498761 PMC11617501

[29] Arfaei Chitkar SS Mohaddes Hakkak HR Saadati H Hosseini SH Jafari Y Ganji R. The effect of mobile-app-based instruction on the physical function of female patients with knee osteoarthritis: a parallel randomized controlled trial. BMC Womens Health. (2021) 21(1):333. 10.1186/s12905-021-01451-w 34521400 PMC8439076

[30] Azma K RezaSoltani Z Rezaeimoghaddam F Dadarkhah A Mohsenolhosseini S. Efficacy of tele-rehabilitation compared with office-based physical therapy in patients with knee osteoarthritis: a randomized clinical trial. J Telemed Telecare. (2018) 24(8):560–5. 10.1177/1357633X17723368 28771070

[31] Khazaei R Maleklou F Bodaghabadi Z Tavana MM Kluzek S Sharafi SE. Developing an 8-week, tele-education weight control and exercise programme, and evaluating its effects on weight and pain reduction in patients with obesity and knee osteoarthritis: a double-blinded randomised clinical trial. Musculoskeletal Care. (2024) 22(3):e1926. 10.1002/msc.1926 39123329

[32] Alasfour M Almarwani M. The effect of innovative smartphone application on adherence to a home-based exercise programs for female older adults with knee osteoarthritis in Saudi arabia: a randomized controlled trial. Disabil Rehabil. (2022) 44(11):2420–7. 10.1080/09638288.2020.1836268 33103499

[33] Nambi G Elayaperumal S Eswaramoorthi V Muthukrishnan R. Effect of tele-rehabilitation on health outcomes in overweight and obese elderly women with locomotor disabilities from knee osteoarthritis: a randomised controlled trial. Hong Kong Physiother J. (2026) 46(1):83–96. 10.1142/S101370252650006X 42016045 PMC13092434

[34] Bennell KL Lawford BJ Keating C Brown C Kasza J Mackenzie D. Comparing video-based, telehealth-delivered exercise and weight loss programs with online education on outcomes of knee osteoarthritis: a randomized trial. Ann Intern Med. (2022) 175(2):198–209. 10.7326/M21-2388 34843383

[35] Hinman RS Nelligan RK Campbell PK Li P Lamb KE Stratulate S. Mobile app to support home exercise adherence in knee osteoarthritis: a randomized clinical trial. Osteoarthritis Cartilage. (2026) 34(4):631–40. 10.1016/j.joca.2025.12.012 41418994

[36] Dieter V Martus P Seißler D Serna-Higuita LM Janssen P Krauss I. Effectiveness of the self-directed mHealth exercise intervention re.flex in patients with knee osteoarthritis: randomized controlled trial. J Med Internet Res. (2025) 27:e71558. 10.2196/71558 41066658 PMC12510439

[37] Weber F Kloek C Stuhrmann S Blum Y Grüneberg C Veenhof C. Usability and preliminary effectiveness of an app-based physical activity and education program for people with hip or knee osteoarthritis - a pilot randomized controlled trial. Arthritis Res Ther. (2024) 26(1):83. 10.1186/s13075-024-03291-z 38600607 PMC11005282

[38] Song Y Reifsnider E Chen Y Wang Y Chen H. The impact of a theory-based mHealth intervention on disease knowledge, self-efficacy, and exercise adherence among ankylosing spondylitis patients: randomized controlled trial. J Med Internet Res. (2022) 24(10):e38501. 10.2196/38501 36264605 PMC9634515

[39] Zhu D Zhao J Wu T Zhu B Wang M Han T. Effects of a computer vision-based exercise application for people with knee osteoarthritis: randomized controlled trial. JMIR Mhealth Uhealth. (2025) 13:e63022. 10.2196/63022 40354624 PMC12088618

[40] Aily JB De Noronha M Approbato Selistre LF Ferrari RJ White DK Mattiello SM. Face-to-face and telerehabilitation delivery of circuit training have similar benefits and acceptability in patients with knee osteoarthritis: a randomised trial. J Physiother. (2023) 69(4):232–9. 10.1016/j.jphys.2023.08.014 37684147

[41] Baker K LaValley MP Brown C Felson DT Ledingham A Keysor JJ. Efficacy of computer-based telephone counseling on long-term adherence to strength training in elderly patients with knee osteoarthritis: a randomized trial. Arthritis Care Res (Hoboken). (2020) 72(7):982–90. 10.1002/acr.23921 PMC10521167 31074576

[42] Cuperus N Hoogeboom TJ Kersten CC Den Broeder AA Vliet Vlieland TPM Van Den Ende CHM. Randomized trial of the effectiveness of a non-pharmacological multidisciplinary face-to-face treatment program on daily function compared to a telephone-based treatment program in patients with generalized osteoarthritis. Osteoarthritis Cartilage. (2015) 23(8):1267–75. 10.1016/j.joca.2015.04.007 25887365

[43] Doumen M De Meyst E Bertrand D Pazmino S Piessens M Joly J. A mobile app to support self-management and remotely monitor disease impact in rheumatoid arthritis: the randomized controlled AEGORA trial. Rheumatology (Oxford). (2025) 64(5):2505–14. 10.1093/rheumatology/keae638 39576683

[44] Knudsen LR Ndosi M Hauge E-M Lomborg K Dreyer L Aaboe S. Effectiveness of a novel digital patient education programme to support self-management of early rheumatoid arthritis: a randomized controlled trial. Rheumatology (Oxford). (2024) 63(9):2547–56. 10.1093/rheumatology/keae177 38498833

[45] Rodríguez Sánchez-Laulhé P Biscarri-Carbonero Á Suero-Pineda A Luque-Romero LG Barrero García FJ Blanquero J. The effects of a mobile app-delivered intervention in people with symptomatic hand osteoarthritis: a pragmatic randomized controlled trial. Eur J Phys Rehabil Med. (2023) 59(1):54–64. 10.23736/S1973-9087.22.07744-9 PMC10035439 36633498

[46] Runhaar J Luijsterburg P Dekker J Bierma-Zeinstra SMA. Identifying potential working mechanisms behind the positive effects of exercise therapy on pain and function in osteoarthritis; a systematic review. Osteoarthritis Cartilage. (2015) 23(7):1071–82. 10.1016/j.joca.2014.12.027 25865391

[47] Zeng C-Y Zhang Z-R Tang Z-M Hua F-Z. Benefits and mechanisms of exercise training for knee osteoarthritis. Front Physiol. (2021) 12:794062. 10.3389/fphys.2021.794062 34975542 PMC8716769

[48] Jackson T Xu T Jia X. Arthritis self-efficacy beliefs and functioning among osteoarthritis and rheumatoid arthritis patients: a meta-analytic review. Rheumatology (Oxford). (2020) 59(5):948–58. 10.1093/rheumatology/kez219 31211379

