# Supplementary material for: The association between telemedicine and patient outcomes in osteoarthritis and inflammatory arthritis: a systematic review and meta-analysis of randomized controlled trials
Source: Front Med (Lausanne). 2026 Oct 14;13:1897145. doi: 10.3389/fmed.2026.1897145 (PMC13713862; doi:10.3389/fmed.2026.1897145)
Supplement: Supplementary file 1 [file Datasheet1.docx]

**Supplementary materials**

**Table S1.** Literature Search Strategy

Pubmed-1630

((("Telemedicine"[Mesh]) OR ((((((((((((((((((((Virtual Medicine) OR (Tele-Referral)) OR (Tele Referral)) OR (Tele-Referrals)) OR (Mobile Health)) OR (mHealth)) OR (Telehealth)) OR (eHealth)) OR (Telecare)) OR (Tele-Care)) OR (Tele Care)) OR (Telerehabilitation)) OR (Telephone)) OR (Smartphone)) OR (Mobile app)) OR (Web-based)) OR (Internet-based)) OR (Video)) OR (Remote)) OR (Digital health))) AND (("Arthritis"[Mesh]) OR ((((((((((((((((Osteoarthritis) OR (Knee osteoarthritis)) OR (Hip osteoarthritis)) OR (Hand osteoarthritis)) OR (Rheumatoid arthritis)) OR (Ankylosing spondylitis)) OR (Spondyloarthritis)) OR (Psoriatic arthritis)) OR (Gout)) OR (Juvenile idiopathic arthritis)) OR (Inflammatory arthritis)) OR (Arthritides)) OR (Polyarthritis)) OR (Polyarthritides)) OR (Oligoarthritis)) OR (Oligoarthritides)))) AND (Random*)

Embase-2961 (Via OVID, https://www.ovid.com/)

((Telemedicine or (Virtual Medicine or Tele-Referral or Tele Referral or Tele-Referrals or Mobile Health or mHealth or Telehealth or eHealth or Telecare or Tele-Care or Tele Care or Telerehabilitation or Telephone or Smartphone or Mobile app or Web-based or Internet-based or Video or Remote or Digital health)) and (Arthritis or (Osteoarthritis or Knee osteoarthritis or Hip osteoarthritis or Hand osteoarthritis or Rheumatoid arthritis or Ankylosing spondylitis or Spondyloarthritis or Psoriatic arthritis or Gout or Juvenile idiopathic arthritis or Inflammatory arthritis or Arthritides or Polyarthritis or Polyarthritides or Oligoarthritis or Oligoarthritides)) and Random*).af.

Cochrane-1904 (Via OVID, https://www.ovid.com/)

((Telemedicine or (Virtual Medicine or Tele-Referral or Tele Referral or Tele-Referrals or Mobile Health or mHealth or Telehealth or eHealth or Telecare or Tele-Care or Tele Care or Telerehabilitation or Telephone or Smartphone or Mobile app or Web-based or Internet-based or Video or Remote or Digital health)) and (Arthritis or (Osteoarthritis or Knee osteoarthritis or Hip osteoarthritis or Hand osteoarthritis or Rheumatoid arthritis or Ankylosing spondylitis or Spondyloarthritis or Psoriatic arthritis or Gout or Juvenile idiopathic arthritis or Inflammatory arthritis or Arthritides or Polyarthritis or Polyarthritides or Oligoarthritis or Oligoarthritides)) and Random*).af.

WOS-1485

(((Telemedicine) OR ((((((((((((((((((((Virtual Medicine) OR (Tele-Referral)) OR (Tele Referral)) OR (Tele-Referrals)) OR (Mobile Health)) OR (mHealth)) OR (Telehealth)) OR (eHealth)) OR (Telecare)) OR (Tele-Care)) OR (Tele Care)) OR (Telerehabilitation)) OR (Telephone)) OR (Smartphone)) OR (Mobile app)) OR (Web-based)) OR (Internet-based)) OR (Video)) OR (Remote)) OR (Digital health))) AND ((Arthritis) OR ((((((((((((((((Osteoarthritis) OR (Knee osteoarthritis)) OR (Hip osteoarthritis)) OR (Hand osteoarthritis)) OR (Rheumatoid arthritis)) OR (Ankylosing spondylitis)) OR (Spondyloarthritis)) OR (Psoriatic arthritis)) OR (Gout)) OR (Juvenile idiopathic arthritis)) OR (Inflammatory arthritis)) OR (Arthritides)) OR (Polyarthritis)) OR (Polyarthritides)) OR (Oligoarthritis)) OR (Oligoarthritides)))) AND (Random*) (Topic)

WHO ICTRP (International Clinical Trials Registry Platform)-231

(((Telemedicine) OR ((((((((((((((((((((Virtual Medicine) OR (Tele-Referral)) OR (Tele Referral)) OR (Tele-Referrals)) OR (Mobile Health)) OR (mHealth)) OR (Telehealth)) OR (eHealth)) OR (Telecare)) OR (Tele-Care)) OR (Tele Care)) OR (Telerehabilitation)) OR (Telephone)) OR (Smartphone)) OR (Mobile app)) OR (Web-based)) OR (Internet-based)) OR (Video)) OR (Remote)) OR (Digital health))) AND ((Arthritis) OR ((((((((((((((((Osteoarthritis) OR (Knee osteoarthritis)) OR (Hip osteoarthritis)) OR (Hand osteoarthritis)) OR (Rheumatoid arthritis)) OR (Ankylosing spondylitis)) OR (Spondyloarthritis)) OR (Psoriatic arthritis)) OR (Gout)) OR (Juvenile idiopathic arthritis)) OR (Inflammatory arthritis)) OR (Arthritides)) OR (Polyarthritis)) OR (Polyarthritides)) OR (Oligoarthritis)) OR (Oligoarthritides)))) AND (Random*)

**Table S2.** Results of Egger’s regression tests for publication bias assessment

| **Outcome** | **No. of studies** | **Intercept coefficient** | **SE** | **t value** | **P value** | **95% CI** |
| --- | --- | --- | --- | --- | --- | --- |
| Pain scores | 12 | −3.493 | 1.762 | −1.98 | 0.076 | −7.418 to 0.433 |
| Quality of life | 11 | 2.976 | 1.880 | 1.58 | 0.148 | −1.277 to 7.228 |
| WOMAC scores | 10 | −3.703 | 2.610 | −1.42 | 0.194 | −9.722 to 2.316 |

Note. SE, standard error; CI, confidence interval; WOMAC, Western Ontario and McMaster Universities Osteoarthritis Index.

**Table S3.** GRADE evidence profile for the main outcomes

| **No. of RCTs** | **Outcomes** | **SMD [95%CI]** | ***I*^2^; P value** | **Risk of bias** | **Inconsistency** | **Indirectness** | **Imprecision** | **Publication bias** | **Plausible confounding** | **Magnitude of effect** | **Dose-response gradient** | **GRADE** |
| --- | --- | --- | --- | --- | --- | --- | --- | --- | --- | --- | --- | --- |
| 12 | Pain score | -0.67 [-1.01, -0.34] | 82%; P <0.00001 | Serious risk | Serious inconsistency | No serious indirectness | No serious imprecision | Strongly suspected | Would not reduce effect | No | No | Very low |
| 11 | Quality of life | 0.42 [0.12, 0.71] | 78%; P <0.00001 | Serious risk | Serious inconsistency | No serious indirectness | Serious imprecision | Undetected | Would not reduce effect | No | No | Very low |
| 10 | WOMAC score | -0.72 [-1.16, -0.29] | 88%; P <0.00001 | Serious risk | Serious inconsistency | No serious indirectness | No serious imprecision | Undetected | Would not reduce effect | No | No | low |
| 5 | Self-efficacy | 0.47 [0.06, 0.89] | 84%; P <0.0001 | Serious risk | Serious inconsistency | No serious indirectness | Serious imprecision | Not assessed | Would not reduce effect | No | No | Very low |

Note. RCT, randomized controlled trial; WOMAC, Western Ontario and McMaster Universities Osteoarthritis Index; CI, confidence interval; SMD, standardized mean difference; GRADE, Grading of Recommendations Assessment, Development and Evaluation.

Figure S1. Risk-of-bias graph for the included randomized controlled trials.

Figure S2. Risk-of-bias summary for the included randomized controlled trials.

Figure S3. Forest plots showing the effects of telemedicine interventions compared with control conditions on KOOS scores, TUG scores, RADAI scores, and PCS scores in patients with arthritis: (a) KOOS scores; (b) TUG scores; (c) RADAI scores; and (d) PCS scores.

**a**

**b**

**c**

Figure S4. Subgroup analyses of the effects of telemedicine interventions on pain scores, quality of life, and self-efficacy stratified by disease type (osteoarthritis vs. inflammatory arthritis): (a) pain scores; (b) quality of life; and (c) self-efficacy.

Figure S5. Funnel plots for publication bias assessment: (a) pain scores; (b) quality of life; (c) WOMAC scores.

Figure S6. Leave-one-out sensitivity analyses for main outcomes: (a) pain scores; (b) quality of life; (c) WOMAC scores; (d) self-efficacy.

**a**

**b**

**c**

Figure S7. Subgroup analyses of the effects of telemedicine interventions on pain scores, quality of life, and WOMAC scores stratified by intervention duration (<8 weeks, 8 weeks, and >8 weeks) after excluding the study by Nambi et al. (2026): (a) pain scores; (b) quality of life; and (c) WOMAC scores.
